# Zinner Syndrome Associated With a Neuroendocrine Tumor of the Prostate Gland and Contralateral Seminal Vesicle: A Case Report

**DOI:** 10.7759/cureus.115459

**Published:** 2026-08-30

**Authors:** Awini Barwari, Josef Finsterer

**Affiliations:** 1 Neurology, Neurology and Neurophysiology Center, Vienna, AUT

**Keywords:** malignancy, neuroendocrine tumor, radical prostatectomy, seminal vesicle, zinner syndrome

## Abstract

Zinner syndrome is a rare congenital malformation of the urogenital system characterized by the triad of unilateral renal agenesis, ipsilateral seminal vesicle cyst, and ipsilateral ejaculatory duct obstruction. A variant of Zinner syndrome is present when, instead of a seminal vesicle cyst, there is agenesis of the ipsilateral seminal vesicle. Although Zinner syndrome has been associated with urogenital neoplasms in some cases, to our knowledge, no case of a patient with Zinner syndrome and a contralateral neuroendocrine tumor (NET) has been previously described.

The patient is a 26-year-old man who developed urinary retention due to a tumor of the right seminal vesicle infiltrating the prostate and obstructing the prostatic urethra. Abdominal and bladder MRI showed dysgenesis of the left kidney, seminal vesicle, and ejaculatory duct, as well as a mildly contrast-enhancing mass of the right seminal vesicle infiltrating the adjacent prostate. Gallium-68 PET-CT showed increased somatostatin receptor (SSTR) expression in the right seminal vesicle. Histologically, it was a well-differentiated NET with infiltrates of epithelial tumor cells that were positive for chromogranin, synaptophysin, and CD56. Chromogranin was also increased in the serum. The patient underwent radical prostatectomy, and it was decided not to proceed with chemotherapy or radiotherapy.

Zinner syndrome may be associated with a contralateral NET of the genitourinary tract. Therefore, patients with Zinner syndrome should be regularly followed up to avoid overlooking a co-occurring malignancy.

## Introduction

Zinner syndrome is a rare congenital malformation of the urogenital system characterized by a triad of unilateral renal agenesis, ipsilateral seminal vesicle cyst, and ipsilateral ejaculatory duct obstruction [1]. A variant of Zinner syndrome is present when, instead of a seminal vesicle cyst, there is agenesis of the ipsilateral seminal vesicle. The embryological basis of Zinner syndrome lies in early maldevelopment of the Wolffian duct (mesonephric duct) during the first trimester of pregnancy (between the 4th and 13th weeks of gestation) [1]. Zinner syndrome is usually asymptomatic until the onset of sexual activity. At this point, the seminal vesicles dilate and cysts form. Affected individuals may then experience perineal or pelvic pain, painful ejaculation, dysuria, urgency, or urinary retention. Blood in the urine or semen may also occur, and patients frequently experience recurrent infections. As the seminal vesicle cysts progressively enlarge, palpable masses may develop in the lower abdomen, which can cause pain and bladder obstruction. Zinner syndrome is the cause of infertility in approximately half of patients. Although Zinner syndrome may be associated with urogenital neoplasms in rare cases [2-14], to our knowledge, no case of a patient with Zinner syndrome and a contralateral neuroendocrine tumor (NET) has yet been described. Causes of a NET in the urogenital tract can include smoking, advanced age, chronic inflammation, hormonal influences, and genetic predisposition. The association between Zinner syndrome and NET is clinically relevant, as the tumor can metastasize and must be detected and treated as quickly as possible.

## Case presentation

The patient is a 26-year-old man (183 cm tall, 73 kg) with a variant of Zinner syndrome, diagnosed based on congenital agenesis of the left kidney, left seminal vesicle, and left ejaculatory duct. There was no family history of urological diseases, and the parents were not consanguineous. The patient was asymptomatic until the age of 26. He then developed an unusual urge to urinate two to 10 minutes after complete bladder emptying, as well as postvoid dribbling. Relative hypospermia and suspected retrograde ejaculation were present. Cystoscopy revealed stenosis of the prostatic urethra due to external compression. Therefore, a urethral incision was performed, and a biopsy was taken. Histological examination of the biopsy revealed invasive urothelial carcinoma (pT2a). Radical prostatectomy with cystectomy and neobladder reconstruction was recommended.

Since the patient was uncertain about the proposed procedure and recommended therapy, he requested a reassessment and a second opinion. Blood tests were unremarkable except for chromogranin A (128.5 ng/mL (normal range: 0-76.3 ng/mL) and 407 ng/mL, respectively) (Table 1). Prostate-specific antigen (PSA) and neuron-specific enolase (NSE) levels were within normal limits (Table 1). MRI of the bladder showed a mildly contrast-enhancing mass in the right seminal vesicle with infiltration of the adjacent prostate (Figure 1). Additionally, an enlarged lymph node was identified below the right iliac bifurcation. MRI of the kidneys and lower abdomen showed agenesis of the left kidney as well as the absence of the left seminal vesicle and left ejaculatory duct (Figure 1). Gallium-68 PET-CT showed increased SSTR expression in the right seminal vesicle (Figure 2). No evidence of an additional primary or metastatic lesion was identified in organs, lymph nodes, or bones showing SSTR-targeted tracer uptake. To rule out a primary gastrointestinal or bronchial tumor, a CT scan of the lungs and abdomen, as well as a gastroscopy and colonoscopy, were performed; all findings were normal.

**Table 1:** Abnormal blood test results for the index patient three months before surgery and shortly before and after radical prostatectomy CRP, C-reactive protein; HD, hospital day; ND: not done; PSA, prostate-specific antigen; RL, reference limits; NSE, neuron-specific enolase

Parameter	RL	HD-82	HD1	HD3	HD4	HD7	HD+10
Erythrocytes	4.2-5-5 T/L	5.61	5.2	4.0	3.9	4.3	ND
Hemoglobin	14-18 g/dL	16.1	15	11.5	11.4	12.5	ND
Hematocrit	42-51%	46.9	44	33.6	33.9	36.8	ND
Thrombocytes	150-400 G/L	231	192	114	122	177	ND
CRP	0.0-4.9 mg/L	0.1	0.3	77	142.9	22.56	ND
PSA	0.00-3.10 ng/mL	0.11	0.16	ND	ND	ND	ND
Chromogranin A	0.0-76.3 ng/mL	407.0	128.5	ND	ND	ND	33.7
NSE	0.0-16.3 µg/L	11.4	9.0	ND	ND	ND	12.4

**Figure 1:**
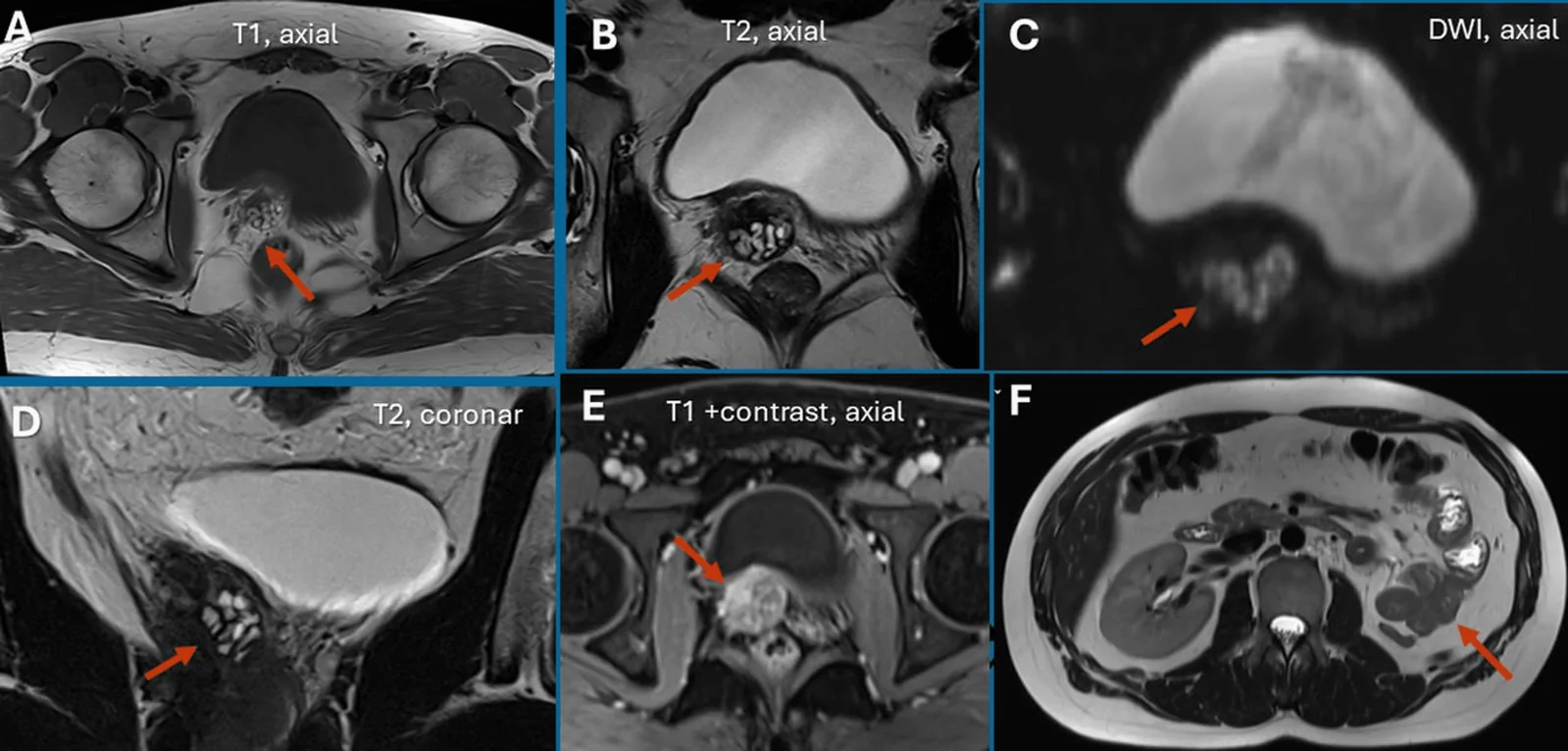
MRI of the prostate and bladder demonstrating a mildly contrast-enhancing mass in the right seminal vesicle (arrows): T1-weighted axial image (A), T2-weighted axial image (B), DWI axial image (C), T2-weighted coronal image (D), and contrast-enhanced T1-weighted axial image (E). MRI of the kidneys and lower abdomen demonstrates agenesis of the left kidney and absence of the left seminal vesicle and left ejaculatory duct. The left renal space is occupied by a loop of bowel (F) DWI, diffusion-weighted imaging

**Figure 2:**
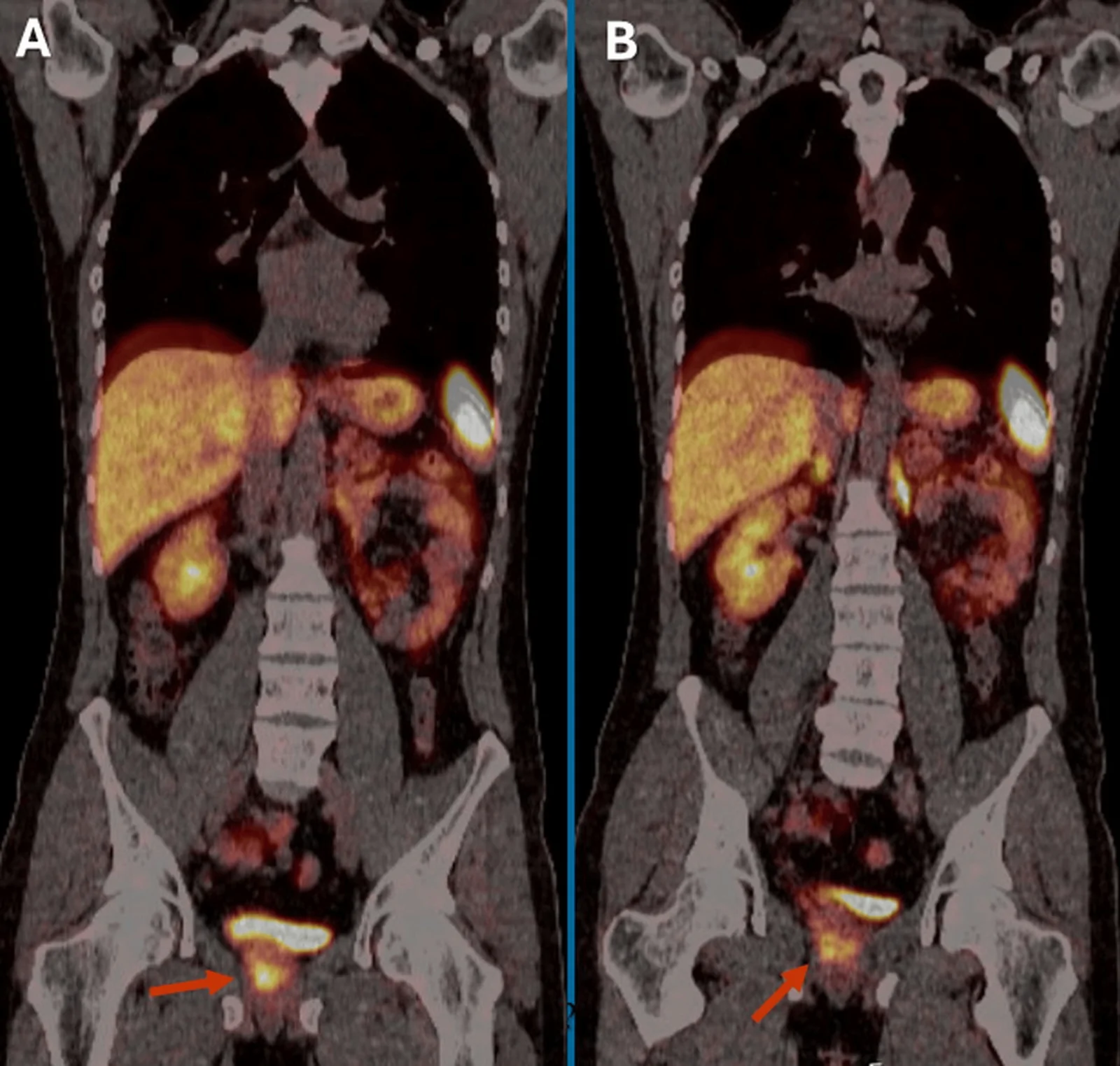
Whole-body PET-CT with gallium-68 shows tracer uptake with an SUVmax of 12.8 in the region of the right seminal vesicle. The mass is in contact with the base of the bladder (A and B)

Following a second biopsy of the tumor, the histological diagnosis was revised, revealing a well-differentiated NET (cTNM (clinical Tumor, Node, Metastasis)) originating from the right seminal vesicle or prostate. Specifically, fibromuscular stroma and sparsely embedded prostatic glands were found, and the tissue was permeated with nerve fibers. Additionally, infiltrates of epithelial neoplasia were present, consisting partly of cribriform and partly of cord-like tumor cell clusters with sparse, poorly demarcated cytoplasm and monomorphic nuclei with coarse chromatin and no nucleoli. Mitoses were barely visible. No necrosis or high-grade atypia was evident. The Ki-67 proliferation index was 3%, supporting the diagnosis of a well-differentiated, low-proliferative NET. Immunophenotyping of the cells was positive for AE1/3 and the endocrine markers chromogranin, synaptophysin, CD56, and INSM1, but negative for CK20, CK7, p40, TTF-1, NKX3.1, inhibin, S100, and SOX10. Cytogenetic analysis revealed a normal karyotype.

The patient underwent robot-assisted radical prostatectomy with parailiacal lymph node dissection. Histological analysis revealed a well-differentiated NET of the prostate and right seminal vesicle, tumor infiltration of the right pelvic wall, and metastasis to one of three lymph nodes in the region of the right external iliac artery. The right vas deferens, urinary bladder, preprostatic tissue, four lymph nodes in the region of the left external iliac artery, and the apical part of the prostate were free of tumor. According to TNM (Tumor, Node, Metastasis) and Union for International Cancer Control (UICC) classification, the tumor was staged as pT3b N1(1/7) cM0 R1 L1 V1 Pn1. The "TruSight Oncology 500 DNA" test, a comprehensive NGS-based (next-generation sequencing) laboratory test for the genomic profiling of tumors, showed a low tumor mutational burden (0.79 mutations/Mb) and microsatellite stability (1.65% unstable loci). No genetic aberrations were detected.

Postoperatively, however, the patient complained of numbness in both feet immediately after waking from the seven-hour general anesthesia. Clinical neurological examination revealed absent Achilles tendon reflexes, bilateral hypoesthesia to touch and pain, and pallial hypoesthesia in the same distribution. Nerve conduction velocity measurements of the peroneal and tibial nerves were normal; however, no nerve action potential could be recorded from either sural nerve. The sensory disturbances were thus attributed to compression of both sural nerves, as the patient's knees were slightly but persistently bent during the operation due to the placement of a roll under the popliteal fossa. Postoperatively, the chromogranin A level normalized (Table 1). Following several tumor board consultations, the preliminary decision was made not to administer postoperative chemotherapy or radiotherapy.

## Discussion

The patient presented here is of interest due to the unusual combination of Zinner syndrome and an infiltrating NET of the contralateral seminal vesicle. Although several benign and malignant urogenital neoplasms [2-14] (e.g., papillary adenomas [4], papillary adenocarcinomas [5], testicular carcinomas [3], mucinous adenocarcinomas [6], squamous cell carcinomas of the seminal vesicle [11], renal cell carcinomas [12], or paragangliomas of the bladder [9]) have been reported in association with Zinner syndrome, the occurrence of a contralateral well-differentiated NET involving the prostate and seminal vesicle has not, to our knowledge, been previously reported. This case therefore highlights an unusual diagnostic and therapeutic challenge in a patient with a congenital Wolffian duct anomaly. Whether the NET in this patient is causally related to Zinner syndrome remains uncertain. Although several urogenital malignancies have previously been reported in patients with Zinner syndrome [2-14], the available evidence is insufficient to establish a causal association.

The second interesting point is that the original histological diagnosis of urothelial carcinoma had to be revised to NET. NETs may be misdiagnosed as other malignant neoplasms when the appropriate immunohistochemical markers are not included in the diagnostic workup, particularly in anatomically unusual locations. NETs are generally divided into poorly differentiated tumors, called neuroendocrine carcinomas, and well-differentiated tumors, called NETs [15]. NETs often show organoid cell proliferation with glandular or rosette-like development, trabecular patterns, and irregular nest formation [15]. Typically, the tumor stroma is sparse and has prominent blood vessels. Occasionally, amyloid stroma, calcifications with psammoma bodies, and a palisade-like cell arrangement near the nest periphery are observed. The tumor cells are typically epithelioid and densely packed, although spindle-shaped and dispersive cells have also been observed [15]. The medium-sized cells have uniformly round to oval nuclei with barely visible nucleoli and abundant cytoplasm. Due to their characteristic fine-to-coarse-grained chromatin, the nuclei exhibit the typical "salt and pepper" appearance [15]. Since the histology of NETs determines the treatment decision, a correct diagnosis is of crucial importance.

## Conclusions

In summary, this case demonstrates that Zinner syndrome can also present as a variant with agenesis of the ipsilateral seminal vesicle instead of a seminal vesicle cyst and that it can be associated with a contralateral NET of the seminal vesicle or prostate. The tumor can infiltrate the right pelvic wall and parailiac lymph nodes. Treatment consists of radical prostatectomy. Patients who experience an unusual urge to urinate shortly after voiding should promptly undergo a thorough urological examination. Patients with Zinner syndrome should undergo regular follow-up examinations to avoid overlooking a possible malignancy on the contralateral side. Zinner syndrome should be considered when there is lower back pain, painful ejaculation, blood in the semen, urinary problems, or infertility, and when unilateral renal and vas deferens agenesis and ipsilateral seminal vesicle agenesis or cyst are present in conjunction with a urogenital tumor on the contralateral side.
